# Prospective outcome analysis of multiple sclerosis cases reveals candidate prognostic cerebrospinal fluid markers

**DOI:** 10.1371/journal.pone.0287463

**Published:** 2023-06-20

**Authors:** Elif Everest, Ugur Uygunoglu, Melih Tutuncu, Alper Bulbul, Umut Inci Onat, Mehmetcan Unal, Timucin Avsar, Sabahattin Saip, Ugur Bilge, Eda Tahir Turanli, Aksel Siva

**Affiliations:** 1 Department of Molecular Biology and Genetics, Faculty of Science and Letters, Istanbul Technical University, Istanbul, Turkey; 2 Department of Neurology, Cerrahpasa School of Medicine, Istanbul University-Cerrahpasa, Istanbul, Turkey; 3 Department of Biostatistics and Bioinformatics, Institute of Health Sciences, Acibadem University, Istanbul, Turkey; 4 Department of Molecular Biology and Genetics, Faculty of Engineering and Natural Sciences, Acibadem University, Istanbul, Turkey; 5 Department of Medical Biology, Faculty of Medicine, Basic Medical Sciences, Bahcesehir University, Istanbul, Turkey; 6 Department of Biostatistics and Medical Informatics, Faculty of Medicine, Akdeniz University, Antalya, Turkey; 7 Graduate School of Natural and Applied Sciences, Molecular and Translational Biomedicine Program, Acibadem University, Istanbul, Turkey; Faculty of Medicine, University of Belgrade, SERBIA

## Abstract

**Background:**

Predicting the long-term disability outcomes of multiple sclerosis (MS) cases is challenging.

**Objective:**

We prospectively analysed our previous MS cohort with initial cerebrospinal fluid (CSF) proteomics data to reveal disability markers after 8.2±2.2 years of follow-up.

**Methods:**

Patients with regular follow-up visits were assigned into two groups: those with an age-related MS severity (ARMSS) score ≥5 (unfavourable course group, N = 27) and ARMSS score <5 (favourable course group, N = 67). A machine learning-based algorithm was applied to reveal candidate poor prognosis-associated initial CSF proteins, which were measured in an independent MS cohort (verification group, N = 40) by ELISA. Additionally, the correlation of initial clinical and radiological parameters with long-term disability was analysed.

**Results:**

CSF alpha-2-macroglobulin (P = 0.0015), apo-A1 (P = 0.0016), and haptoglobin (P = 0.0003) protein levels, as well as cerebral lesion load (>9 lesions) on magnetic resonance imaging, gait disturbance (P = 0.04), and bladder/bowel symptoms (P = 0.01) were significantly higher in the unfavourable course group than in the favourable course group. Optic nerve involvement evident on initial magnetic resonance imaging (P = 0.002) and optic neuritis (P = 0.01) were more frequent in the favourable course group.

**Conclusion:**

The herein identified initial CSF protein levels, in addition to the clinical and radiological parameters at disease onset, have predictive value for long-term disability in MS cases.

## Introduction

Multiple sclerosis (MS) is a chronic neuro-inflammatory and neurodegenerative disease of the central nervous system with a complex pathophysiology and clinical course. Numerous studies have revealed potential prognostic markers, including magnetic resonance imaging (MRI) parameters [1–3] and disease- and progression-related cellular pathways, most of which are related to immune system dysregulation [4], blood-brain barrier disruption [5], oligodendrocyte death [6], and axonal loss [7]. At disease onset, it is important to decide the type of treatment in a patient-specific manner based on the overall clinical, MRI, immunological, neurophysiological, and all other prognostic findings of each patient. However, although many clinical and radiological studies have been conducted, the time of progression cannot be reliably predicted based on these parameters, highlighting the need for strong biological markers for improved treatment decision-making. In this context, various candidate prognostic serum and cerebrospinal fluid (CSF) biomarkers have been proposed for MS, such as neurofilament light chain [8] and glial fibrillary acid protein [9]. However, to the best of our knowledge, there is no large-scale prospective clinical analysis using initial CSF proteomics data of an MS cohort with long-term clinical follow-up. Such prospective analyses are warranted to lend credence to the proposed candidate biomarkers and identify novel molecules with prognostic values, as well as to help further clarify the pathogenesis of the disease.

Previously, we applied a CSF proteomics approach using 2D-gel electrophoresis and mass spectrophotometry and identified differentially expressed proteins and affected cellular pathways among MS cases with different clinical phenotypes and non-MS controls [10]. Here, using a machine learning-based algorithm, we re-analysed the initial proteomics data of this MS cohort, identifying potential prognostic biomarkers of poor prognosis after a follow-up period of 8.2±2.2 years. Additionally, we examined the clinical and MRI characteristics at disease onset that are associated with long-term disability outcomes. Finally, we measured CSF levels of the identified candidate protein markers in an independent MS cohort with a 6.4±2.6-year follow-up period after the diagnosis.

## Materials and methods

### Prospective MS cohort and clinical data collection

A total of 94 MS patients were included in this study. The patients were diagnosed according to the McDonald 2005 or 2010 criteria [11, 12] based on the date of their diagnosis and were retrospectively confirmed that all fulfilled the McDonald 2017 criteria [13]. All patients were diagnosed and have been followed in the Neurology Department of Istanbul University-Cerrahpasa, Cerrahpasa School of Medicine. The Ethics Committee of Istanbul University-Cerrahpasa approved the study (approval number: 930211, 2009), and each individual provided an informed consent form prior to inclusion in the study. The inclusion criteria were patients with 1) at least 5 years of follow-up and 2) proteomic data obtained in our previous study [10]. The following information was recorded for each patient: age, sex, follow-up period, initial diagnosis, diagnosis at the last visit, Expanded Disability Status Scale (EDSS) score at the time of spinal tap, EDSS score at the last visit, age-related MS severity (ARMSS) score based on the last follow-up, oligoclonal band pattern, IgG index, lesion localisation at disease onset, and symptom at disease onset. Assessment of lesion localisation included cerebral lesion load (the number of T2 lesions ≤9 vs. >9), optic nerve involvement, brainstem lesions, spinal cord lesions, and multifocal involvement. Symptoms at disease onset were categorised into sensory symptoms, motor symptoms, gait disturbance, optic neuritis, diplopia and other brainstem symptoms, bladder/bowel symptoms, and Lhermitte’s sign. Based on prognosis, the patients were divided into two groups: the favourable course group consisted of patients with an ARMSS score lower than 5 (N = 67), and patients with an ARMSS score equal to or higher than 5 were assigned to the unfavourable course group (N = 27). Each parameter was compared between the two groups to reveal possible associations of demographic, clinical, and radiological findings with prognosis.

### Machine learning-based genetic algorithm

A machine learning-based genetic algorithm was used to search for algorithmic rules that can be predictive of MS prognosis based on CSF proteomic data obtained at the time of diagnosis consisting of 151 differentially expressed proteins identified through 2D gel electrophoresis and mass spectrometry [10]. Genetic algorithm is a search and optimisation technique that is based on principles of Darwinian evolution [14] and is suitable for problems with a large number of continuous as well as categorical variables. The algorithm works by starting with randomly generated hypotheses to explain a target variable, applying mutation and crossover to these hypotheses and testing each one of them with a user-defined fitness function. We used a genetic algorithm software package developed by Bilge et al., which allows pre-processing, visualising datasets, and selecting different fitness functions [15]. The system converts continuous-valued inputs into a desired number of categories by grouping them into an equal number of samples in each category or using cut-off values provided by users. The programme automatically selects and allows users to set the total number of hypotheses and the number of hypotheses that survive to the next generation, as well as mutation and crossover rates. Based on the study design, higher sensitivity or specificity can be achieved by selecting the appropriate fitness function for the genetic algorithm. In this study, the system was used to generate several rules that differentiate the favourable and unfavourable course patient groups, yielding varying sensitivity and specificity values. For each rule, we identified patients who were not covered by the rule either as false positives or false negatives.

### Verification MS cohort and ELISA assays

CSF levels of A2M, apo-AI, and CD36 proteins that were identified by the genetic algorithm approach were measured in an independent verification MS cohort by ELISA. Additionally, CSF haptoglobin level was measured since there was a significant difference in its initial CSF level between the favourable and unfavourable groups of the prospective MS cohort and due to its relevance to MS as shown by previous studies. The inclusion criteria for the verification cohort were patients 1) whose CSF samples were collected at the time of the first episode before the initiation of any treatment at least 5 years before the experiments; 2) who had been followed up until the experiments to allow the calculation of ARMSS scores based on the last follow-up; and 3) who agreed to participate in the study. Patients whose CSF samples were analysed in our previous study were excluded from the analysis. Among eligible patients, we selected 40 patients with the longest follow-up period (6.4±2.6 years): 20 in the favourable course group (ARMSS score <5) and 20 in the unfavourable course group (ARMSS score ≥5). Proteins were measured using ELISA kits (A2M, ab108883, Abcam; apo-AI, ab108803, Abcam; CD36, ab267614, Abcam; haptoglobin, E-EL-H6078, Elabscience) according to the manufacturers’ protocols. All samples were measured in duplicate. Protein levels were compared between the groups by Student’s t-test.

### Statistical analyses

Continuous variables are presented as means and standard deviations (SD), and categorical variables are presented as numbers and percentages. For continuous variables, differences in mean values between the study groups were compared using the Student’s t-test. For categorical variables, differences in proportions between the study groups were compared using Fisher’s exact test. P<0.05 was considered statistically significant. All statistical analyses were performed using GraphPad Prism 8 Version 8.4.3, and bar plots were drawn using matplotlib library in Python 3.9.

## Results

### Clinical characteristics associated with long-term prognosis

**Fig 1:**
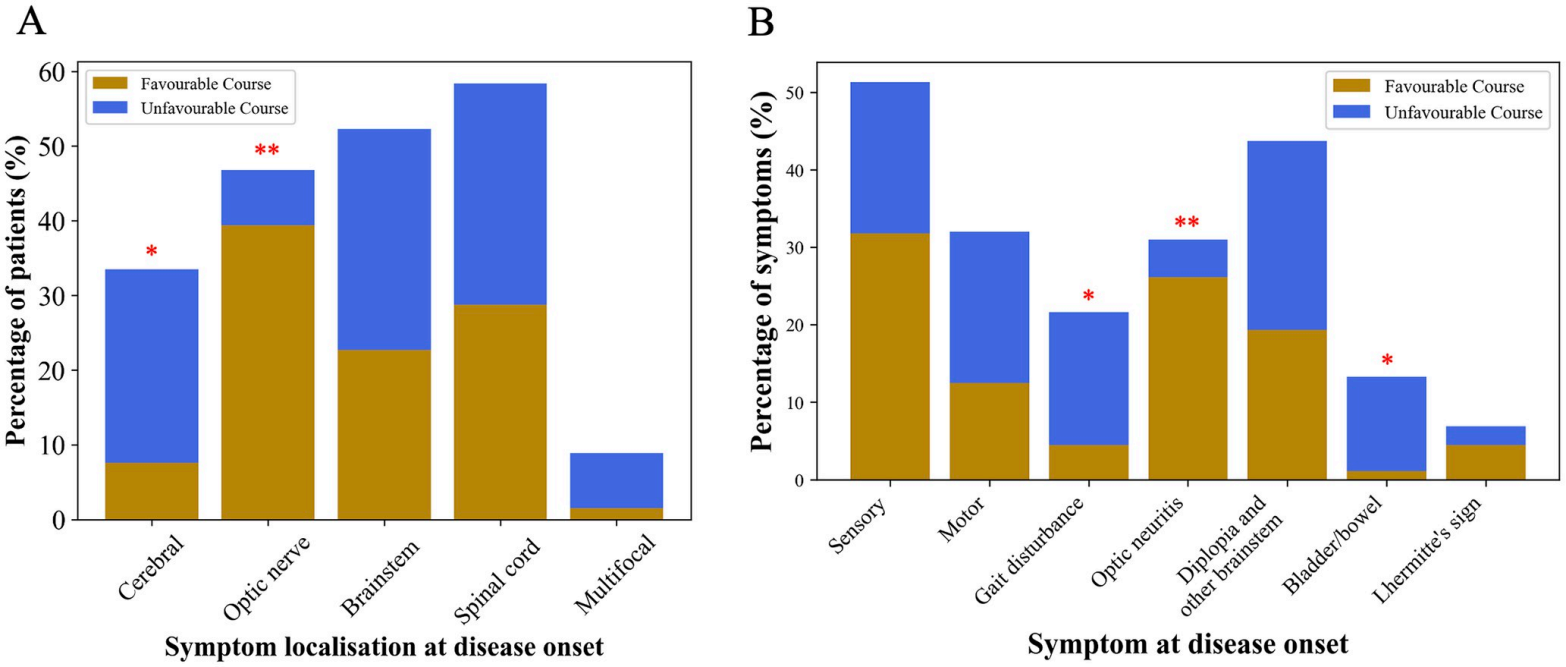
(A) The percentages of patients based on the initial lesion localisation and (B) the percentages of the initial symptom types in the favourable (ARMSS score <5) and unfavourable course (ARMSS score ≥5) groups of the prospective MS cohort. Fisher’s exact test. *, P<0.05; **, P<0.01.

Demographic and clinical characteristics of the prospective MS cohort are given in Table 1. The mean follow-up period was 8.2±2.2 years. There were no significant differences in age, sex, or follow-up period between the favourable and unfavourable course groups. Although statistically insignificant, patients with poor prognosis tended to have a higher oligoclonal band pattern positivity (96% vs. 77.4%, P = 0.06), while the mean IgG index was similar between the groups (P = 0.21). The EDSS score at the time of spinal tap and at the last visit, as well as the ARMSS score calculated based on the last follow-up, were significantly higher in the unfavourable course group (P<0.0001 for all, indicating that the two groups were well-divided based on the disability statuses of the patients). The percentage of patients with high cerebral lesion load at disease onset was significantly higher in the unfavourable course group than in the favourable course group (25.9% vs. 7.6%, respectively, P = 0.04), while the percentage of those with optic nerve involvement evident on initial MRI was significantly higher in the favourable course group than in the unfavourable course group (39.4% vs. 7.4%, respectively, P = 0.002, Table 1 and Fig 1A). Each symptom at disease onset was compared between the groups; optic neuritis was associated with a favourable outcome (P = 0.004), while gait disturbance (P = 0.04) and bladder/bowel symptoms (P = 0.01) were higher in the unfavourable course group (Table 1, Fig 1B). After diagnosis, 80.8% of the patients in the favourable course group and 100% of the patients in the unfavourable course group received a disease-modifying therapy (P = 0.026). This difference is expected and compatible with clinical practise, considering that the initial clinical and radiological characteristics of the patients who later fall into the unfavourable course group would lead to a higher frequency of immediate disease-modifying therapy initiation by physicians. Disease-modifying drugs do not have an effect on CSF protein levels measured in this study since all CSF samples were collected prior to treatment initiation.

**Table 1:** Demographic and clinical characteristics of the prospective MS cohort.

	Favourable Course Group (N = 67)	Unfavourable Course Group (N = 27)	P value
**Age, years (mean ± SD)**	41.9 ± 9.9	44.2 ± 9.8	0.31
**Female sex, number (%)**	48 (71.6%)	17 (63%)	0.46
**Follow-up, years (mean ± SD)**	8.04 ± 2.15	8.63 ± 2.65	0.26
**Initial diagnosis, number**	SAMS, 48	SAMS, 6	
	RRMS, 19	RRMS, 9	
	PPMS, 0	PPMS, 10	
	SPMS, 0	SPMS, 2	
**Diagnosis at last visit, number**	SAMS, 21	SAMS, 0	
	RRMS, 46	RRMS, 12	
	PPMS, 0	PPMS, 10	
	SPMS, 0	SPMS, 5	
**EDSS at the time of spinal tap (mean ± SD)**	0.61 ± 0.76	2.13 ± 1.84	<0.0001*
**Last EDSS (mean ± SD)**	0.94 ± 1.01	4.8 ± 1.95	<0.0001*
**ARMSS based on the last follow-up (mean ± SD)**	1.79 ± 1.26	7.22 ± 1.53	<0.0001*
**Oligoclonal band positivity, number (%)**	48 (77.4%)	24 (96%)	0.06
**IgG index**	0.89 ± 0.62	1.21 ± 0.87	0.21
**Disease-modifying treatment initiation at onset, number (%)**	42/52 (80.8%)	24/24 (100%)	0.026*
**Symptom localization at disease onset, number (%)**	Cerebral, 5 (7.6%)	Cerebral, 7 (25.9%)	0.04*
	Optic nerve, 26 (39.4%)	Optic nerve, 2 (7.4%)	0.002*
	Brainstem, 15 (22.7%)	Brainstem, 8 (29.6%)	0.6
	Spinal cord, 19 (28.8%)	Spinal cord, 8 (29.6%)	1
	Multifocal, 1 (1.5%)	Multifocal, 2 (7.4%)	0.2
**Symptom at disease onset, number (%)**	Sensory, 28 (31.8%)	Sensory, 8 (19.5%)	0.21
	Motor, 11 (12.5%)	Motor, 8 (19.5%)	0.3
	Gait disturbance, 4 (4.5%)	Gait disturbance, 7 (17.1%)	0.04*
	Optic neuritis, 23 (26.1%)	Optic neuritis, 2 (4.9%)	0.004*
	Diplopia and other brainstem symptoms, 17 (19.3%)	Diplopia and other brainstem symptoms, 10 (24.4%)	0.5
	Bladder/bowel, 1 (1.1%)	Bladder/bowel, 5 (12.2%)	0.01*
	Lhermitte’s sign, 4 (4.5%)	Lhermitte’s sign, 1 (2.4%)	1

ARMSS, age-related multiple sclerosis severity; CIS, clinically isolated syndrome; EDSS, Expanded Disability Status Scale; MS, multiple sclerosis; PPMS, primary progressive MS; RIS, radiologically isolated syndrome; RRMS, relapsing-remitting MS; SAMS, single-attack MS; SD, standard deviation; SPMS, secondary progressive MS;

*, statistically significant.

SAMS refers to patients who presented with CIS at the first inflammatory demyelinating episode of the central nervous system. This initial group of patients with CIS was retrospectively evaluated, and those who fulfilled the McDonald 2017 criteria were diagnosed and included as SAMS. Other brainstem symptoms include dysarthria, dysphagia, and vestibular/cochlear symptoms. Favourable outcome is defined as an ARMSS score < 5 and unfavourable outcome as an ARMSS score ≥ 5.

### Genetic algorithm approach reveals candidate CSF proteins associated with long-term prognosis

We found several rules explaining poor prognosis using the initial CSF proteomics data of the prospective MS cohort. Two rules with varying sensitivity and specificity values are detailed in Table 2. We have selected rule 2 to verify its predictive value of MS prognosis in an independent MS cohort, although we obtained a number of other rules having better sensitivity but containing proteins that were not properly identified by mass spectrometry (e.g., uncharacterized peptides) in the former study, or there were no available protein measurement assays for some proteins involved in those rules. Therefore, we tested rule 2 since we were able to identify and quantify all the proteins involved in the rule, which also showed a strong specificity.

**Table 2:** Rules and their predictive properties for the long-term disease outcome identified by the genetic algorithm.

Rule 1	Rule 2
ARMSS> = 5.0	ARMSS<5.0
IF {	IF {
*C3bCfb< = 1*.*0 AND*	*CD36< = 1*.*0 AND*
*A2M< = 2*.*5 AND*	*A2M>2*.*5 AND*
*ATF7< = 1*.*0 AND*	*ApoA1< = 1*.*0*
*PRBP< = 1*.*0 AND*	}
*Haptoglobin< = 1*.*0 AND*	
*PDS5B< = 1*.*0 AND*	
*Myosin< = 1*.*0*	
}	
Rule Performance	Rule Performance
TP = 15, T = 20	TP = 20, T = 43
FP = 1, F = 43	FP = 0, F = 20 sensitivity = 46.51%
sensitivity = 75% specificity = 97.67%	specificity = 100%
accuracy = 90.48%	accuracy = 63.49%
PPV = 93.75%	PPV = 100%
NPV = 89.36%	NPV = 46.51% kappa = 35.57%
kappa = 76.78%	ROC AUC = 73.26%
ROC AUC = 86.34%	

Protein values shown in the rules represent the fold change of corresponding proteins between the patient groups with ARMSS≥5 and ARMSS<5 in the prospective MS cohort. TP, true positive; FP, false positive; sen, sensitivity; spe, specificity; acc, accuracy; ppv, positive predictive value; npv, negative predictive value; ROC, receiver operating characteristic; AUC, area under the curve; C3bCfb, chain F, crystal structure of complement C3b in complex with factor B; A2M, alpha-2-macroglobulin; ATF7, cyclic AMP-dependent transcription factor ATF-7; PRBP, plasma retinol binding protein; PDS5B, human androgen-induced prostate proliferative shutoff associated protein (AS3); Myosin, human skeletal mRNA for myosin heavy chain light meromyosin region; ApoA1, apolipoprotein A1.

### CSF protein measurement of candidate biomarkers in a verification MS cohort

**Fig 2:**
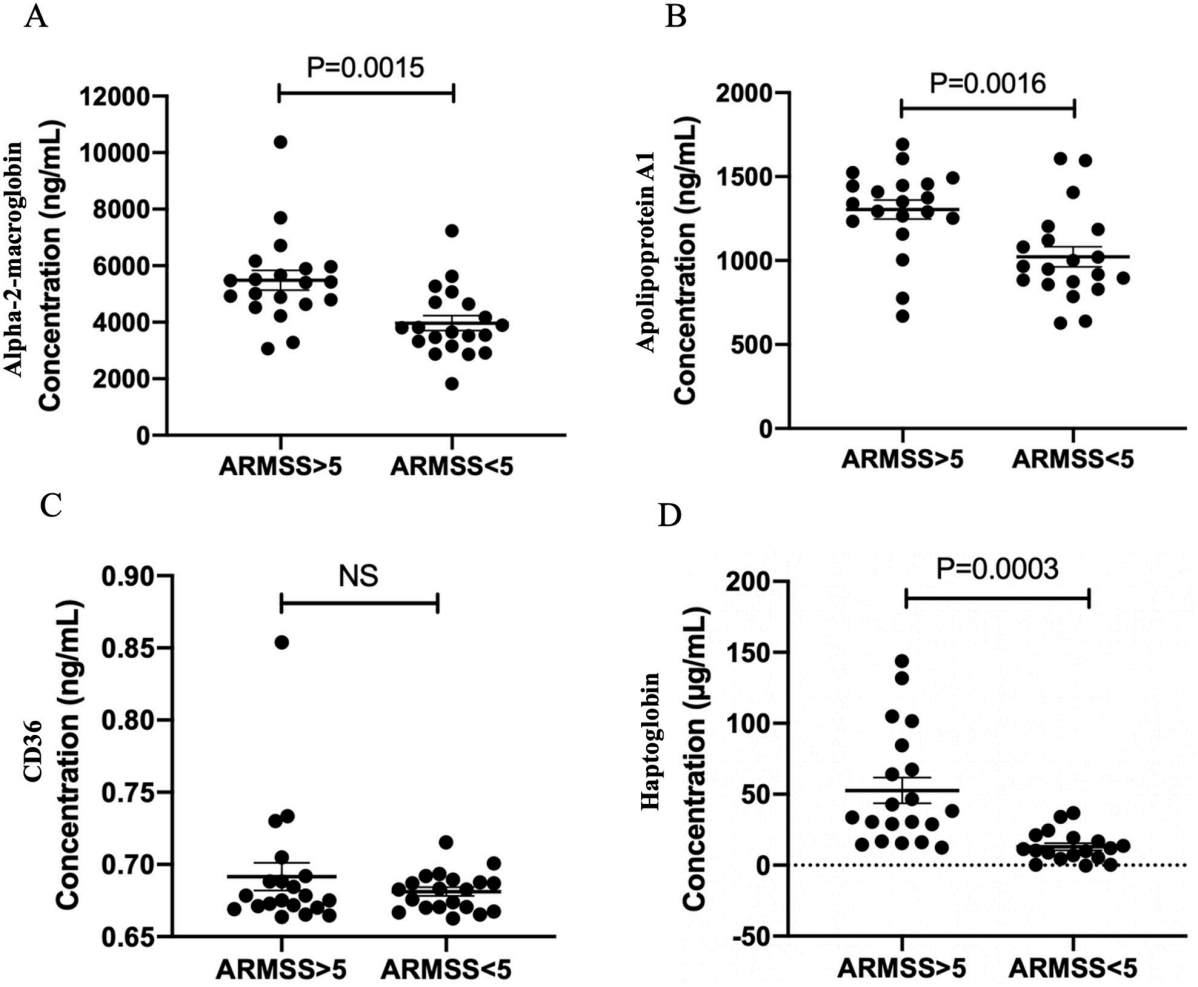
Differences in cerebrospinal fluid (A) alpha-2-macroglobin, (B) apolipoprotein A1, (C) CD36, and (D) haptoglobin levels between the favourable and unfavourable course groups in the verification MS cohort, measured by enzyme-linked immunosorbent assays. Two-tailed Student’s t-test; P<0.05, significant. ARMSS, age-related MS severity.

The mean follow-up period of the verification cohort was 6.4±2.6 years. Patients in the unfavourable course group had significantly higher ARMSS scores than those in the favourable course group based on the last follow-up (7.0±1.7 vs. 1.7±0.9, P<0.0001). CSF A2M and apo-AI levels were significantly higher in patients with unfavourable course than in patients with favourable course (P = 0.0015 and P = 0.0016, respectively, Fig 2A and 2B), while CD36 levels were not different between the two groups (Fig 2C). The difference in CD36 level was still insignificant when three outlier values were excluded from the analysis. CSF haptoglobin level was not significantly different between the two groups; however, when two outlier values were excluded from the analysis, the difference became significant (P = 0.0003, Fig 2D).

## Discussion

In the present study, we provide clinical and candidate prognostic biomarkers identified by a prospective analysis of an MS cohort that has been followed for nearly a decade following their CSF proteomics analysis in a previous study [10]. Currently, long-term prognostic predictors of MS course in the early disease stages are mainly clinical and radiological findings. Although available data can provide insights into a general prediction for each patient’s prognosis to some extent, there is an evident need for a laboratory tool that can more reliably measure the probability of a patient developing the progressive phase during the disease course. Given that the efficacy, risk, and side effect profiles of each drug vary and that treatment options have been increasing in MS treatment, such laboratory biomarkers that help in treatment decisions based on the progression risk of patients are highly desirable. Moreover, MS treatment can be ceased in some patients who do not develop the progressive disease after a clinical age cut-off in light of those clinical and radiological parameters in a patient-specific manner. This decision should also be supported by a laboratory assessment that can further indicate that progression is no longer a risk factor for the patient in these cases. In this context, the measurement of serum and CSF neurofilament light chain levels has been increasingly studied and seems to be a strong candidate biomarker of ongoing and possibly upcoming neurodegeneration [8]. Recently, glial fibrillary acid protein has also been shown to be a promising candidate prognostic marker in MS [9]. Here, using a genetic algorithm, we provide additional candidate biomarkers of poor prognosis that can be measured in CSF samples of patients at the diagnosis stage. The genetic algorithm approach is highly suitable for medical data mining tasks as opposed to black box approaches, where neural networks are able to learn from data but cannot provide explanations. Genetic algorithm rules can be tailored for higher sensitivity or specificity depending on the requirement, and the rules can be further analysed in terms of false negative and false positive numbers. Regarding the disability measure, we grouped the patients based on their ARMSS scores. ARMSS was developed by Manouchehrinia et al. using disability data from three population-based cohorts and ranking EDSS scores based on the patients’ age at the time of assessment [16]. We used the ARMSS measure rather than the last EDSS or MS Severity Score (MSSS) to obtain more comprehensive disability status information instead of a snapshot view since the ARMSS score, calculated based on age, was shown to be a slightly better disability indicator than the MSSS, calculated based on disease duration [17]. Given the presence of the subclinical MS stage with varying periods, we preferred the age-based disability measure since disease duration might be a somewhat vague parameter for such calculations.

Among the identified candidate proteins, A2M, a protease inhibitor and cytokine transporter, has been previously implicated in MS. Tenorio-Laranga et al. showed that serum activated A2M level was significantly lower in clinically isolated syndrome (CIS), relapsing-remitting MS (RRMS), primary progressive MS (PPMS), and secondary progressive MS (SPMS) cases compared with healthy controls [18]. On the other hand, in a proteomic analysis by Bijak et al., blood platelet A2M was found to be increased in SPMS patients compared with healthy controls [19], in accordance with our current findings showing a higher initial CSF A2M level in patients with worsened disability over time. Moreover, transformed A2M level was found to be elevated in serum samples of PPMS and SPMS cases compared with controls with other neurological diseases, while total A2M levels did not show a significant difference [20]. There are also a number of earlier studies showing differences in isoelectric points of CSF, serum, and plasma A2M protein in MS or MS subgroups. Serum A2M level was also shown to be elevated in preclinical Alzheimer’s disease (AD) and associated with a significantly higher risk of progression to clinical symptoms, reflecting early neuronal injury [21]. By expression-based analyses, the authors further revealed that *SLIT2*, which is involved in the regulation of blood-brain barrier permeability, was one of the genes in the A2M network. Considering that the highly multifunctional A2M protein can influence a broad range of biological processes and binds to a wide range of ligands, including cytokines, we believe that an altered level or function of A2M has importance in MS pathophysiology and progression, possibly through changes in the blood-brain barrier permeability. Although the studies of A2M in MS differ in the sample type (i.e., CSF, plasma, serum, or blood platelets) and protein form (i.e., native, activated, or total), study results indicate that the protein may have functional importance in MS, and elucidating the molecular mechanisms in which different A2M forms are involved may have importance in MS pathophysiology and prognosis.

Apo-AI is a major protein component of high-density lipoproteins, mainly involved in lipid metabolism, with other functions such as immunity, inflammation, and apoptosis. Although Apo-AI is synthesised in the liver and intestine, it enters the brain through the choroid plexus and is thus associated with many neurological conditions. Previous research has shown that higher serum apo-AI levels correlate with lower grey matter and cortical volume loss and a lower conversion rate to SPMS during the 5-year follow-up period [22, 23]. On the contrary, we found a higher CSF apo-AI level in patients with an unfavourable disease course in this study. It was shown that apo-AI exerts anti-inflammatory properties by inhibiting the pro-inflammatory cytokines IL-1b and TNFa [24]. Lower CSF apo-AI levels in patients with a favourable course in our cohort may be linked to a lower degree of suppression of these pro-inflammatory cytokines by apo-AI, resulting in a more inflammatory disease phase as expected in relapsing-active MS. The discrepancy may be due to the different types of samples studied; further research is needed to elucidate the association of apo-AI with MS progression.

Haptoglobin is produced by hepatocytes and binds to free haemoglobin, mainly playing an oxidative stress blocker role [25], with a diverse range of functions including antioxidation and regulation of the immune system. In our study, CSF haptoglobin showed an increase in the unfavourable course group, suggesting a prognostic indication for worsening disability. In a previous study, a higher CSF haptoglobin level was observed in neuromyelitis optica (NMO) patients compared with MS cases and patients with other neurological diseases (OND), and CSF haptoglobin level and index were also significantly positively correlated with EDSS in NMO cases, similar to that observed in our MS cohort [26]. While CSF haptoglobin levels were similar between MS and OND in this analysis, another study revealed a significant increase in CSF haptoglobin level in MS compared with OND [27]. Given the anti-inflammatory and anti-oxidative functions of haptoglobin, initial CSF haptoglobin levels in patients with worsened disability over time might have increased in response to increased oxidative stress—known to be strongly associated with neurodegeneration—as a compensatory mechanism. Increased CSF haptoglobin levels have also been observed in traumatic brain injury, AD, and Guillain-Barre syndrome, suggesting that increased CSF haptoglobin is not specific to MS pathophysiology but may have long-term prognostic value for MS.

One limitation of our study is the relatively small sample size due to the absence of the clinical data of some of the patients from the original cohort who have stopped attending their routine follow-up appointments. Second, we were unable to test better genetic algorithm rules regarding sensitivity, either because some proteins were not properly identified by mass spectrometry in our previous study or because there were no specific assays available to measure their absolute values. Lastly, the correlation between CSF protein levels and clinical parameters other than ARMSS scores could not be performed since the sample sizes of related patient subgroups were small and thus would lead to unreliable results.

In conclusion, we provide novel candidate CSF protein biomarkers—as well as clinical and radiological parameters that are compatible with the literature—at the initial stages of clinical MS that may predict long-term disability outcomes in MS patients. Confirming these data in larger MS cohorts, preferably with longer follow-up periods and in different sample types of patients, as well as correlating them with other suggested prognostic predictors, such as novel MRI findings, optical coherence tomography, and serological biomarkers (e.g., serum neurofilament light chain and GFAP), may contribute to improved treatment decision-making in MS.
